# Effect of sitagliptin on the echocardiographic parameters of left ventricular diastolic function in patients with type 2 diabetes: a subgroup analysis of the PROLOGUE study

**DOI:** 10.1186/s12933-017-0546-2

**Published:** 2017-05-11

**Authors:** Hirotsugu Yamada, Atsushi Tanaka, Kenya Kusunose, Rie Amano, Munehide Matsuhisa, Hiroyuki Daida, Masaaki Ito, Hiroyuki Tsutsui, Mamoru Nanasato, Haruo Kamiya, Yasuko K. Bando, Masato Odawara, Hisako Yoshida, Toyoaki Murohara, Masataka Sata, Koichi Node, for the PROLOGUE Study Investigators

**Affiliations:** 1 Department of Cardiovascular Medicine, Tokushima University Hospital, 2-50-1 Kuramoto, Tokushima, Japan; 2 Department of Cardiovascular Medicine, Saga University, 5-5-1 Nabeshima, Saga, Japan; 3 Department of Diabetes Therapeutics and Research Center, Tokushima University, Tokushima, Japan; 4 Department of Cardiovascular Medicine, Juntendo University Graduate School of Medicine, Tokyo, Japan; 5 Department of Cardiology and Nephrology, Mie University Graduate School of Medicine, Tsu, Japan; 6 Department of Cardiovascular Medicine, Faculty of Medical Sciences, Kyushu University, Fukuoka, Japan; 7 Cardiovascular Center, Japanese Red Cross Nagoya Daini Hospital, Nagoya, Japan; 8 Division of Cardiology, Japanese Red Cross Nagoya Daiichi Hospital, Nagoya, Japan; 9 Department of Cardiology, Nagoya University Graduate School of Medicine, Nagoya, Japan; 10 Department of Diabetes, Endocrinology, Metabolism and Rheumatology, Tokyo Medical University, Tokyo, Japan; 11 Clinical Research Center, Saga University, Saga, Japan

**Keywords:** Sitagliptin, T2DM, Echocardiography, Diastolic function, NT-proBNP

## Abstract

**Background:**

Diabetes is associated closely with an increased risk of cardiovascular events, including diastolic dysfunction and heart failure that leads to a shortening of life expectancy. It is therefore extremely valuable to evaluate the impact of antidiabetic agents on cardiac function. However, the influence of dipeptidyl peptidase 4 inhibitors on cardiac function is controversial and a major matter of clinical concern. We therefore evaluated the effect of sitagliptin on echocardiographic parameters of diastolic function in patients with type 2 diabetes as a sub-analysis of the PROLOGUE study.

**Methods:**

Patients in the PROLOGUE study were assigned randomly to either add-on sitagliptin treatment or conventional antidiabetic treatment. Of the 463 patients in the overall study, 115 patients (55 in the sitagliptin group and 60 in the conventional group) who had complete echocardiographic data of the ratio of peak early diastolic transmitral flow velocity (E) to peak early diastolic mitral annular velocity (e′) at baseline and after 12 and 24 months were included in this study. The primary endpoint of this post hoc sub-analysis was a comparison of the changes in the ratio of E to e′ (E/e′) between the two groups from baseline to 24 months.

**Results:**

The baseline-adjusted change in E/e′ during 24 months was significantly lower in the sitagliptin group than in the conventional group (−0.18 ± 0.55 vs. 1.91 ± 0.53, p = 0.008), irrespective of a higher E/e′ value at baseline in the sitagliptin group. In analysis of covariance, sitagliptin treatment was significantly associated with change in E/e′ over 24 months (β = −9.959, p = 0.001), independent of other clinical variables at baseline such as blood pressure, HbA1c, and medications for diabetes. Changes in other clinical variables including blood pressure and glycemic parameters, and echocardiographic parameters, such as cardiac structure and systolic function, were comparable between the two groups. There was also no significant difference in the serum levels of N-terminal-pro brain natriuretic peptide and high-sensitive C-reactive protein between the two groups during the study period.

**Conclusions:**

Adding sitagliptin to conventional antidiabetic regimens in patients with T2DM for 24 months attenuated the annual exacerbation in the echocardiographic parameter of diastolic dysfunction (E/e′) independent of other clinical variables such as blood pressure and glycemic control.

*Trial registration* UMIN000004490 (University Hospital Medical Information Network Clinical Trials). https://upload.umin.ac.jp/cgi-open-bin/ctr_e/ctr_view.cgi?recptno=R000005356; registered November 1, 2010

**Electronic supplementary material:**

The online version of this article (doi:10.1186/s12933-017-0546-2) contains supplementary material, which is available to authorized users.

## Background

Type 2 diabetes mellitus (T2DM) is associated closely with an increased risk of cardiovascular (CV) events including heart failure [1, 2]. The prevalence of patients who develop heart failure is greater in diabetic individuals than in non-diabetic individuals, and diabetes is known to be a strong risk factor for the development of heart failure [3]. It has been shown that once individuals with T2DM developed heart failure their 5-year survival rate was 12.5%, a rate considerably lower than in individuals without heart failure [3]. Furthermore, diabetes contributes to a worse outcome in patients with left ventricular (LV) diastolic dysfunction than those with systolic dysfunction [5]. However, intensive glucose-lowering therapy with antidiabetic agents does not always reduce the risk of heart failure [6], with some agents having unfavorable clinical effects on heart failure [7, 8]. Therefore, it is important to evaluate the impact of antidiabetic agents on cardiac function [9, 10].

To date, three randomized controlled trials that focused on major CV outcomes in patients with T2DM treated with either dipeptidyl peptidase-4 (DPP-4) inhibitors or placebo have been reported. Alogliptin in the EXAMIN [11], saxagliptin in the SAVOR-TIMI 53 [12], and sitagliptin in the TECOS [13] all showed non-inferior to placebo to lower the risk of the composite primary endpoint of CV death, myocardial infarction or ischemic stroke. However, in the SAVOR-TIMI 53 trial a 27% increase in the rate of hospital admission for heart failure was found in the group with saxagliptin [14]. Results from meta-analyses of randomized trials also demonstrated that DPP-4 inhibitors were associated with an increased risk of heart failure [15, 16]. In contrast, there was no significant difference in the rate of hospital admissions for heart failure between sitagliptin and placebo groups in the TECOS trial. Taken together, these results show the influence of DPP-4 inhibitors on cardiac function is still a major clinical concern.

The PROLOGUE study (University hospital Medical Information Network Center: ID 000004490) was a prospective multicenter study conducted in Japan to evaluate the inhibitory effect of sitagliptin on the progression of atherosclerosis based on carotid-artery intima-media thickness (IMT) assessed by ultrasonography over a 2-year follow-up period [17, 18]. In this study, echocardiography at baseline and after 12 and 24 months of treatment was an optional examination. In order to elucidate the effect of DPP-4 inhibitor on cardiac function we carried out a sub-study of the PROLOGUE study that investigated the effect of sitagliptin on two-dimensional and Doppler echocardiographic parameters, mainly focusing on left ventricular diastolic function from baseline to 24 months.

## Methods

### Study design

The details of the PROLOGUE study design have been published elsewhere [17]. Briefly, the study was a multicenter, randomized, prospective, open-label, blinded-endpoint trial carried out at 48 institutions in Japan. A total of 463 patients older than 30 years who had T2DM with an HbA1c level of 6.2–9.4% despite conventional treatment with diet, exercise, and/or pharmacologic therapy with oral antidiabetic agents (except incretin-related therapy) for more than 3 months were enrolled in the study between June 2011 and September 2012. Patients with severe heart failure with a New York Heart Association (NYHA) functional classification of III and IV were excluded. The inclusion and exclusion criteria for the study have been published previously [17, 18]. The patients were assigned randomly using a 1:1 ratio to either add-on sitagliptin treatment (sitagliptin group, n = 232) or conventional glucose-lowering treatment (conventional group, n = 231). The primary endpoint of the PROLOGUE study was the change in mean common carotid IMT 24 months after treatment randomization. Echocardiography was performed as an ad hoc examination at baseline and 12 and 24 months after treatment randomization. The ethical committees of each participating institution approved the study protocol, with written informed consent for participation in the study being obtained from all the subjects.

### Study population

**Fig. 1:**
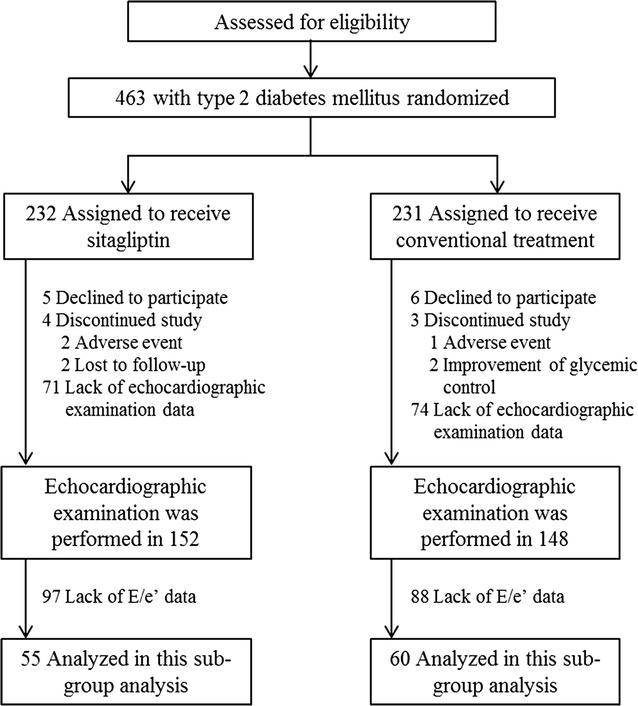
Study participant flow diagram. E/e′: ratio of peak early diastolic transmitral flow velocity (E) to peak early diastolic mitral annular velocity (e′)

Of the 436 participants in the PROLOGUE study, an echocardiographic examination was performed at baseline in 152 patients in the sitagliptin group and 148 patients in the conventional group. The present study analyzed the data of 115 patients (55 patients in the sitagliptin group and 60 patients in the conventional group) who had echocardiographic data of the ratio of peak early diastolic transmitral flow (TMF) velocity (E) to peak early diastolic mitral annular velocity (e′) at baseline and at both 12 and 24 months (Fig. 1).

### Echocardiographic examination

Echocardiography was performed in a standard manner using commercially available ultrasound diagnostic machines with various hemodynamic parameters being measured at each institution. The recordings and measurements were performed in accordance with the guidelines issued by the American Society of Echocardiograph [19]. TMF velocity was recorded from the apical long-axis or four-chamber view. The ratio of the peak early diastolic (E) and the peak atrial systolic (A) TMF velocities was calculated. The deceleration time (DT) of early TMF velocity was also measured. The mitral annular motion velocity pattern was recorded from the apical four-chamber view with the sample volume located at the lateral or septal side of the mitral annulus using pulsed tissue Doppler echocardiography. The mean peak early diastolic mitral annular velocity (e′) in the septal and lateral side was measured, and the ratio of E to e′ (E/e′) then calculated as a marker of LV filling pressure. In addition to these diastolic parameters, routine echocardiographic parameters were also measured and included LV end-diastolic dimensions (LVDd) and LV end-systolic dimensions (LVDs) measured from M-mode or 2-dimensional echocardiogram of the LV. Fractional shortening was calculated as (LVDs—LVds)/LVDdx100. The LV ejection fraction (LVEF) was measured and calculated from the apical two- and four-chamber view using a modified Simpson’s method. LV mass was calculated as reported previously [20]. Relative wall thickness was calculated as two times posterior wall thickness divided by LVDd [21]. All Doppler recordings were performed during an end-expiratory breath hold. The mean values of three consecutive cardiac cycles were used in the analysis. Measurement and interpretation of the echocardiography was performed locally at each institution. The readers were blinded to the patients’ assignment to treatment.

### Laboratory examination

Blood samples were collected at baseline and after 12 and 24 months. The parameters analyzed are listed in Table 1. The serum levels of N-terminal pro-brain natriuretic peptide (NT-proBNP) and high-sensitive CRP were measured in a centralized laboratory (SRL Co. Tokyo, Japan) using an electrochemiluminescence immunoassay (ECLIA) and nephelometry, respectively.

**Table 1:** Clinical characteristics between the two groups

Variables	Baseline			24 months			Least square means of baseline-adjusted changes		
	Sitagliptin (n = 55)	Conventional (n = 60)	p value	Sitagliptin (n = 55)	Conventional (n = 60)	p value	Sitagliptin (n = 55)	Conventional (n = 60)	p value
Age, year	69 ± 8	69 ± 9	0.973						
Gender (male/female)	38/17	39/21	0.641						
Body mass index, kg/m^2^	25.9 ± 3.3	24.8 ± 3.9	0.098	25.5 ± 3.4	24.6 ± 3.8	0.203	−0.10 ± 0.16	−0.13 ± 0.15	0.368
Systolic BP, mmHg	132 ± 17	129 ± 19	0.287	133 ± 16	130 ± 20	0.377	1.70 ± 2.20 (n = 54)	0.34 ± 2.09	0.656
Diastolic BP, mmHg	74 ± 11	71 ± 12	0.090	75 ± 13 (n = 54)	71 ± 11	0.076	1.76 ± 1.47 (n = 54)	−0.54 ± 1.40	0.263
Heart rate, beats/min	70 ± 12	67 ± 12	0.196	71 ± 16	67 ± 10	0.129	3.37 ± 1.49	−2.14 ± 1.43	0.009
Total cholesterol, mmol/L	4.4 ± 0.8	4.6 ± 0.9	0.43	4.4 ± 0.8 (n = 54)	4.4 ± 1.0	0.690	−0.08 ± 0.09 (n = 54)	−0.10 ± 0.08	0.899
HDL cholesterol, mmol/L	1.3 ± 0.3 (n = 55)	1.4 ± 0.4 (n = 58)	0.617	1.3 ± 0.3 (n = 54)	1.4 ± 0.4	0.412	−0.03 ± 0.03 (n = 54)	0.00 ± 0.03 (n = 58)	0.362
Triglycerides, mmol/L	3.1 [2.2–4.5] (n = 54)	3.1 [2.3–4.2]	0.536	2.9 [2.2–5.0] (n = 54)	2.9 [2.1–3.9]	0.151	0.06 ± 0.06 (n = 53)	−0.08 ± 0.05	0.078
Creatinine, μmol/L	71.6 [61.9–86.6]	68.1 [59.2–89.3]	0.496	75.1 [62.8–87.5] (n = 54)	69.0 [61.0–98.1]	0.740	2.65 ± 1.77 (n = 54)	4.42 ± 1.77	0.345
eGFR, mL/min/1.73 m^2^	66.6 ± 15.9	67.3 ± 18.4	0.834	65.1 ± 14.1 (n = 54)	67.1 ± 19.7	0.757	−1.96 ± 1.10 (n = 54)	–3.18 ± 1.04	0.419
Fasting plasma glucose, mmol/L	7.5 ± 1.8 (n = 53)	7.1 ± 1.4	0.135	7.0 ± 1.6 (n = 54)	6.7 ± 1.7 (n = 58)	0.248	−0.41 ± 0.20 (n = 52)	−0.49 ± 0.19 (n = 58)	0.780
HbA1c, %	7.0 ± 0.6	6.9 ± 0.5	0.737	6.5 ± 0.6 (n = 54)	6.6 ± 0.7	0.412	−0.47 ± 0.08 (n = 52)	−0.34 ± 0.07 (n = 57)	0.211
1,5AG, µg/mL	13.9 [8.3–20.3] (n = 52)	15.6 [10.7–22.8] (n = 58)	0.086	16.4 [8.7–22.6]	15.1 [9.6–25.0] (n = 59)	0.747	2.55 ± 0.82 (n = 52)	0.95 ± 0.79 (n = 57)	0.165
NT-proBNP, pg/mL	111.5 [42.1–240.8] (n = 52)	99.6 [52.1–234.9] (n = 58)	0.848	114.5 [51.8–261.9]	114.1 [60.2–323.8] (n = 59)	0.673	0.09 ± 0.08 (n = 52)	0.16 ± 0.08 (n = 57)	0.535
High-sensitive CRP, ng/mL	540 [279–1100] (n = 52)	576 [236–1618] (n = 58)	0.952	478 [232–1150] (n = 55)	478 [199–1590] (n = 59)	0.984	−0.06 ± 0.16 (n = 52)	−0.06 ± 0.15 (n = 57)	0.983
Current smoker, n (%)	8 (17.4) (n = 46)	13 (28.3) (n = 46)	0.321						
Hypertension, n (%)	45 (81.8)	46 (76.7)	0.497						
Dyslipidemia, n (%)	42 (76.4)	42 (70.0)	0.530						
Cerebrovascular disease, n (%)	8 (14.6)	4 (6.7)	0.226						
Cardiovascular disease, n (%)	38 (69.1)	41 (68.3)	0.930						
Chronic heart failure, n (%)	3 (5.5)	7 (11.7)	0.326						
Medications									
ACE inhibitor or ARB	37 (67.3)	42 (70.0)	0.753	39 (70.9)	41 (68.3)	0.764			
β-blocker	12 (21.8)	11 (18.3)	0.641	13 (23.6)	12 (20.0)	0.637			
Diuretic	17 (30.9)	12 (20.0)	0.178	16 (29.1)	15 (25.0)	0.621			
Statin	42 (76.4)	40 (66.7)	0.251	39 (70.9)	39 (65.0)	0.498			
α-Glucosidase inhibitor, n (%)	23 (41.8)	30 (50.0)	0.455	18 (32.7)	38 (63.3)	0.001			
Glinide, n (%)	4 (7.3)	4 (6.7)	0.899	2 (3.6)	5 (8.3)	0.293			
Biguanide, n (%)	9 (16.4)	10 (16.7)	0.965	12 (21.8)	16 (26.7)	0.545			
Sulfonylurea, n (%)	9 (16.4)	12 (20.0)	0.638	4 (7.3)	18 (30.0)	0.002			
Thiazolidinedione, n (%)	11 (20.0)	18 (30.0)	0.283	9 (16.4)	23 (38.3)	0.009			

Data for categorical variables are given as number (%); data for continuous variables given as mean ± standard deviation or median [interquartile range]. Skewed data was calculated after logarithmic translation. In the right column, values are shown as baseline-adjusted least square mean ± standard error

*BP* blood pressure, *HDL* high-density lipoprotein, *eGFR* estimated glomerular filtration rate, *1,5AG* 1,5-anhydroglucitol,1,4-anhydro-d-glucitol, *NT-proBNP* N-terminal pro-brain natriuretic peptide, *CRP* C-reactive protein. *ACE* angiotensin-converting enzyme, *ARB* angiotensin receptor blocker

### Statistical analysis

Data were expressed as mean ± standard deviation for normally distributed variables, median and interquartile range for variables with a skewed distribution, and frequencies (%) for categorical variables. All reported probability values were two-sided with a p value <0.05 considered statistically significant. The percentage changes in the variables during the study period were calculated as (values obtained at 12 or 24 months after treatment randomization—the baseline value)/baseline value. The differences between the two groups were assessed, where appropriate, by either the Student’s *t* test, Mann–Whitney test, or Fisher’s exact test. Variables with a skewed distribution were analyzed in the analysis of covariance after logarithmic conversion. We performed baseline-adjusted and multivariable regression analysis to confirm differences between the two groups. All the analyses were conducted using the JMP software program, version 12.1.0 (SAS Institute Inc., Cary, NC, USA).

## Results

### Clinical characteristics

Table 1 shows a comparison of the clinical characteristics at baseline and at 24 months and baseline-adjusted changes after 24 months of glycemic control between the two patient groups. There was no difference in body mass index and blood pressure between the groups throughout the study, while heart rate was increased in the sitagliptin group at 24 months. Although more than 70% of the subjects had hypertension, blood pressure was well controlled in both groups. Other parameters, such as lipid and renal profiles, were similar in the two groups throughout the study. The incidence of a previous history of CV diseases, including heart failure was not different in the two groups. Although the use of background medications for hypertension, dyslipidemia, or diabetes at baseline was also comparable in the groups, the incidence of some types of antidiabetic agent increased during the treatment period. This was especially apparent in the conventional group, possibly due to many patients achieving the glycemic control goal (HbA1c <6.2%) set in the PROLOGUE study protocol.

### Glycemic control and neurohumoral effects

The levels of fasting plasma glucose, HbA1c, and 1, 5 AG were similar at baseline in the two groups and there were no significant changes in these parameters during the 24 months of treatment between the groups (Table 1). These results indicate similar degrees of improved glycemic control had been achieved. The serum levels of NT-proBNP and high-sensitive CRP were also similar at baseline and after 24 months of treatment.

### Echocardiographic parameters

**Fig. 2:**
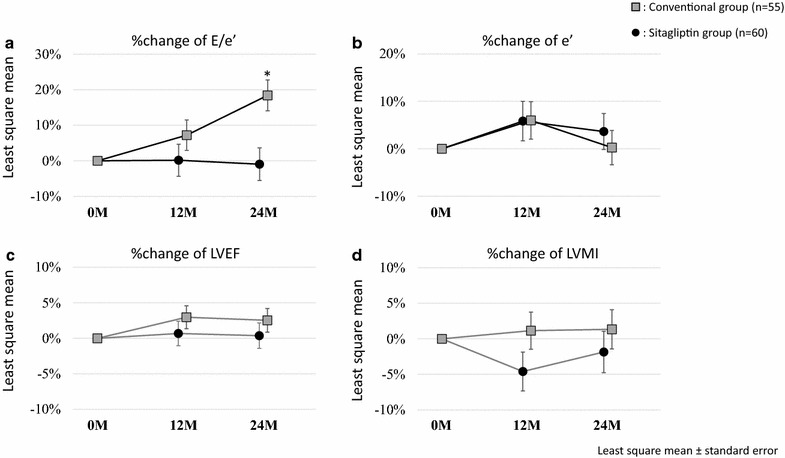
Percentage changes in E/e′, e′, LVEF, and LVMI at 12 and 24 months in the two treatment groups. Each graph shows sex-, age- and baseline-adjusted least square means (±standard error) at 12 and 24 months. The %change values were calculated as (24 or 12 month data-baseline)/baseline. E/e′ at 24 months shows significant difference between the two groups. *E* peak early diastolic transmitral flow velocity, *e′* peak early diastolic mitral annular velocity, *LVEF* left ventricular ejection fraction, *LVMI* left ventricular mass index. *p = 0.002 vs. sitagliptin group

A comparison of echocardiographic parameters at baseline and after 24 months of treatment and baseline-adjusted changes after 24 months of treatment in both groups is shown in Table 2. Although baseline E/e′ was significantly higher in the sitagliptin group than in the conventional group, the baseline-adjusted change in E/e′ during 24 months of treatment was significantly lower in the sitagliptin group than in the conventional group (Fig. 2a). Analysis of covariance showed this difference remained significant (sitagliptin group, −30.9 ± 9.8%/24 months; conventional group, −11.0 ± 9.0%/24 months; p = 0.001, Table 3), even after adjustment for various confounding factors, such as age, sex, baseline systolic blood pressure, baseline HbA1c, history of CV diseases, history of heart failure, baseline medications for diabetes, baseline E/A, baseline LVEF, and baseline LV mass index. Other parameters relevant to diastolic function such as e′, E/A, and DT were similar in the two groups during the 24 months of treatment (Table 2; Fig. 2b). There were also no significant differences in parameters of cardiac structure and systolic function at baseline and 24 months, or baseline-adjusted changes after 24 months of treatment between the two groups (Table 2; Fig. 2c, d).

**Table 2:** Comparisons of echocardiographic parameters at baseline, 24 months and baseline-adjusted changes after 24 months of glycemic control

Variables	Baseline			24 months			Least square means of baseline-adjusted changes		
	Sitagliptin (n = 55)	Conventional (n = 60)	p value	Sitagliptin (n = 55)	Conventional (n = 60)	p value	Sitagliptin (n = 55)	Conventional (n = 60)	p value
TMF-E, cm/s	74.2 ± 32.6	69.3 ± 31.0	0.417	73.1 ± 33.4	73.6 ± 34.3	0.939	−0.85 ± 2.37	4.04 ± 2.27	0.139
e′, cm/s	6.35 ± 1.72	7.03 ± 2.13	0.065	6.60 ± 2.19	6.76 ± 2.11	0.684	0.13 ± 0.23	−0.16 ± 0.22	0.370
E/e′	12.17 ± 5.20	10.34 ± 4.18	0.040	12.19 ± 6.93	12.06 ± 7.06	0.922	−0.18 ± 0.55	1.91 ± 0.53	0.008
TMF-A, cm/s	80.1 ± 20.3 (n = 48)	83.4 ± 21.3 (n = 54)	0.424	82.4 ± 19.3 (n = 48)	88.2 ± 22.9 (n = 55)	0.168	3.06 ± 2.10 (n = 47)	5.73 ± 1.96 (n = 54)	0.354
E/A	0.91 ± 0.52(n = 48)	0.79 ± 0.24 (n = 54)	0.125	0.86 ± 0.35 (n = 48)	0.81 ± 0.31 (n = 54)	0.418	−0.03 ± 0.04 (n = 48)	−0.04 ± 0.04 (n = 54)	0.825
Deceleration time, msec	233.5 ± 65.8 (n = 50)	237.6 ± 67.7 (n = 55)	0.751	238.1 ± 78.1 (n = 52)	240.6 ± 64.6 (n = 58)	0.859	9.53 ± 9.06 (n = 49)	5.38 ± 8.63 (n = 54)	0.741
LV end-diastolic dimension, mm	48.5 ± 5.6	48.6 ± 5.9	0.912	47.5 ± 5.7	47.7 ± 5.3	0.836	−1.04 ± 0.51	−0.91 ± 0.48	0.855
LV end-systolic dimension, mm	31.7 ± 6.1	32.6 ± 7.2	0.462	31.5 ± 7.1	31.6 ± 6.9	0.935	−0.30 ± 0.60	−0.94 ± 0.58	0.447
Fractional shortening, %	35.1 ± 6.7	33.6 ± 7.9	0.279	33.9 ± 13.3	34.4 ± 8.5	0.787	−1.05 ± 1.31	0.64 ± 1.25	0.355
LV ejection fraction, %	63.6 ± 9.6	60.8 ± 10.8	0.153	62.5 ± 9.3	61.9 ± 9.8	0.721	−0.63 ± 0.85 (n = 54)	0.67 ± 0.81	0.273
LV mass, g	173.0 ± 66.6	160.2 ± 56.3	0.266	159.9 ± 49.1	159.2 ± 52.9	0.940	−10.29 ± 4.94	−3.50 ± 4.73	0.324
LV mass index, g/m^2^	101.6 ± 35.0	96.2 ± 30.6	0.383	94.8 ± 26.3	95.6 ± 28.3	0.881	−5.50 ± 2.81	−1.80 ± 2.68	0.343
Relative wall thickness	0.40 ± 0.09	0.38 ± 0.08	0.230	0.41 ± 0.08	0.39 ± 0.08	0.400	0.01 ± 0.01	0.00 ± 0.01	0.886
LA dimension, mm	40.8 ± 6.9 (n = 54)	40.0 ± 7.6 (n = 58)	0.572	39.7 ± 6.9	40.3 ± 6.9 (n = 59)	0.652	−0.87 ± 0.55 (n = 54)	0.25 ± 0.54 (n = 58)	0.151

Data for categorical variables are given as number (%); data for continuous variables given as mean ± standard deviation. In the right column, values are shown as baseline-adjusted least square mean ± standard error

*TMF* transmitral flow velocity, *E* early diastolic velocity, *e′* early diastolic mitral annular velocity, *LV* left ventricular, *LA* left atrial

**Table 3:** Factors associated with change in E/e′ from baseline to 24 months

	Model 1		Model 2		Model 3	
	N = 115, R = 0.26		N = 115, R = 0.27		N = 115, R = 0.55	
	β	p value	β	p value	β	p value
Sitagliptin	−8.910	0.006	−8.887	0.007	−9.959	0.001
E/e′	−0.094	0.889	−0.159	0.820	−0.240	0.716
Male gender			0.674	0.845	0.149	0.966
Age			0.330	0.392	−0.005	0.990
Systolic blood pressure					−0.293	0.095
HbA1c					6.614	0.272
Cardiovascular disease					−5.631	0.142
Chronic heart failure					10.819	0.070
α-glucosidase inhibitor					1.053	0.734
Biguanide					−14.839	0.001
Glinide					−10.778	0.072
Sulfonylurea					−0.567	0.891
Thiazolidinedione					2.159	0.572
LV ejection fraction					0.178	0.602
LV mass index					0.348	<0.001

Model 1 means ANCOVA adjusted for baseline E/e′. Model 2 were adjusted for Model 1 and sex, age. Model 3 were adjusted for Model 2 and systolic blood pressure, HbA1c, cardiovascular disease, chronic heart failure, α-glucosidase inhibitor, bigunaide, glinide, sulfonylurea, thiazolidinedione, LV ejection fraction, LV mass index, whose data were obtained at baseline examination

Abbreviations, see Tables 1 and 2

## Discussion

The present study was a subgroup analysis of the PROLOGUE trial that focused on the effect of sitagliptin on echocardiographic parameters of diastolic function. The key finding of the study was that addition of sitagliptin to conventional diabetic care significantly attenuated the increase in echocardiographic parameters of diastolic function (E/e′), relative to conventional treatment alone. On the other hand, changes in other parameters such as LV size and LVEF did not differ between the two groups. We also found no significant differences in the biomarkers measured during the study. It is known that metabolic disturbances and diabetes are associated closely with cardiac diastolic dysfunction such as diabetic cardiomyopathy, and there is also evidence that patients with diabetes and an increased E/e′ have higher mortality [22, 23]. Given these results, it appears that sitagliptin treatment may have a protective effect on cardiac diastolic function, leading to improved prognosis independent of glycemic control and blood pressure.

Recently we demonstrated a possible effect of sitagliptin on carotid atherosclerosis [18], endothelial function [24], and arterial stiffness [25] using data of the PROLOGUE study. This series of studies did not show beneficial effects of sitagliptin treatment on these variables, relative to conventional glucose-lowering treatment with the exception of incretin-related agents, with better glycemic control being observed in the sitagliptin treatment group. In contrast, there is another report that additional administration of DPP-4 inhibitors, including sitagliptin, to conventional antidiabetic regimes significantly attenuated the progression of carotid IMT [26, 27]. Although the participants’ backgrounds including age, concomitant agents, and severity of diabetes differed between these studies [18], these findings suggest that DPP-4 inhibitors at least cause no harm to the vasculature and are useful for glycemic control in the usual clinical settings. The findings are also consistent with the results of a large CV outcome trial [13]. This led us to investigate the effect of sitagliptin on cardiac function and biomarkers in the current sub-group analysis of the PROLOGUE study data.

DPP-4 inhibitors promote glucose-dependent insulin secretion and suppress glucagon secretion by inhibiting the activity of an enzyme which inactivates endogenous incretin like glucagon-like peptide-1 (GLP-1) and gastric inhibitory polypeptide. This leads to improved postprandial hyperglycemia similar to that seen with the normal physiological response. DPP-4 inhibitors do not cause weight gain and a single administration is unlikely to induce hypoglycemia. It is therefore relatively easy to use DPP-4 inhibitors safely in the elderly and patients with renal dysfunction. To date, the three randomized clinical trials mentioned above have reported non-inferiority, relative to placebo, for CV outcomes in patients with T2DM with high cardiovascular risk or established CV disease [11–13]. In particular, the rate of hospitalization for heart failure was similar between sitagliptin and placebo treatments in the TECOS trial [13], despite the SAVOR-TIMI 53 trial showing that saxagliptin, another DPP-4 inhibitor, significantly increased the hospitalization rate [12]. However, because these trials did not fully investigate CV physiological functions and relevant biomarkers, it proved difficult to determine how DPP-4 inhibitors affected cardiac function. Mechanistic studies using these surrogate markers are therefore required to determine the possible actions of DPP-4 inhibitors on the CV system.

Accumulated evidence suggests that patients with TD2M often exhibit LV diastolic dysfunction and heart failure due to underlying metabolic derangement, such as insulin resistance, independent of hypertension and coronary artery disease (CAD) [28–30]. Recent studies have also demonstrated that DPP-4 activity correlates with cardiac systolic and diastolic dysfunction and remodeling via several molecular pathways, such as increased inflammation and altered angiogenesis [31–34]. Experimental studies have shown that sitagliptin improved survival rate and cardiac function in an ischemia–reperfusion mice model [35] and reduced infarction size in a myocardial infarction mice model [36]. Long-term administration of sitagliptin was also shown to suppress the onset of heart failure in a rat model of heart failure [31]. A meta-analysis of clinical trials also described the advantages of DPP-4 inhibitors on risk reduction in CV events and death compared with other antidiabetic agents [37]. Sitagliptin treatment in T2DM patients with CAD also improved parameters of diastolic function and cardiac dysfunction due to post-ischemic stunning during dobutamine stress echocardiography [38]. In contrast, sitagliptin treatment did not improve systolic function in T2DM patients with ischemic heart failure [39]. Furthermore, Oe et al. [40] reported that sitagliptin treatment in T2DM patients with LV diastolic dysfunction was not associated with improvement in the relevant echocardiographic parameters. As a consequence of these different findings the therapeutic effect of DPP-4 inhibitors on cardiac function remains controversial.

In the present study, adding sitagliptin to usual diabetes treatment significantly attenuated the annual increase in E/e′, suggesting a preventive effect on LV compliance and diastolic dysfunction. However, sitagliptin treatment did not affect other echocardiographic parameters of systolic function and cardiac structures or other clinical variables, such as NT-proBNP, blood pressure, and glycemic control. Comparison with a previous study in which sitagliptin did not improve diastolic dysfunction [40] showed the following differences: (1) all participants in the previous study were diagnosed with diastolic dysfunction at baseline; (2) the treatment period was 6 months vs. 24 months in the PROLOGUE study; (3) the comparator was voglibose vs. any antidiabetic agents except for incretin-related in the PROLOGUE study. While these differences may have affected the results of the studies, the precise mechanisms by which sitagliptin suppressed the increase in diastolic parameter values were not confirmed in our study. As reported previously [40], the increased incidence of concomitant use of thiazolidinediones in the conventional group may have enhanced the acceleration of E/e′ values. Nogueira et al. also reported that beneficial effects in LV diastolic function were observed in T2DM patients on insulin treated with sitagliptin, while the effects were not as apparent in T2DM patients treated with insulin only [41]. That study also reported a possible association between the sitagliptin-mediated improvement in diastolic dysfunction and increase in plasma GLP-1 levels. However, we did not measure this incretin in the current study. It is thought that GLP-1 has a wide spectrum of CV protective effects [42]. In fact, treatment with a GLP-1 agonist, one of the incretin-related agents, was shown to improve diastolic function beyond and independent of glycemic control [43]. Because there remains clinical caution regarding DPP-4 inhibitor-induced heart failure [44, 45], further experimental and clinical research is required to elucidate the precise mechanisms by which DPP-4 inhibitors affect diastolic function and heart failure in patients with T2DM.

### Limitations

The present study was a sub-analysis of the PROLOGUE study. Because echocardiography was a voluntary measurement in the PROLOGUE study and not performed in all participants, the number of patients in this study was small and included only Japanese subjects. Whether or not echocardiography was performed was left to the judgment of each researcher and therefore selection bias could not be fully excluded. The sample size may therefore be underpowered and accordingly the clinical implications may be limited. In addition, the PROLOGUE study recruited patients with and without history of heart failure at baseline, with patients with a NYHA functional classification of III and IV being excluded. Because most patients had no history of heart failure evident at baseline, we did not determine whether there were differences in the effects of sitagliptin on diastolic function between patients with or without heart failure. Further studies on a larger number of subjects are needed to assess whether longer-term DPP-4 inhibitor treatment is safe and has beneficial effect on cardiac function in T2DM patients with or without overt heart failure.

## Conclusions

Our present sub-group analysis from the PROLOGUE study demonstrated that adding sitagliptin to conventional antidiabetic regimens for 24 months in patients with T2DM attenuated the annual exacerbation in the echocardiographic parameter of diastolic dysfunction, E/e′, independent of other clinical variables such as blood pressure and glycemic control. These results suggest that sitagliptin is potentially a beneficial agent for diastolic function in patients with T2DM.

## Additional file

**Additional file 1.** The PROLOGUE Study Investigators.

## Abbreviations

A: peak atrial systolic transmitral flow velocity
CAD: coronary artery disease
CV: cardiovascular
DPP-4: dipeptidyl peptidase 4
DT: deceleration time
E: peak early diastolic transmitral flow velocity
e′: peak early diastolic mitral annular velocity
EF: ejection fraction
GLP-1: glucagon-like peptide-1
IMT: intima-media thickness
LV: left ventricular
LVDd: left ventricular end-diastolic dimensions
LVDs: left ventricular end-systolic dimensions
NT-proBNP: N-terminal pro-brain natriuretic peptide
NYHA: New York Heart Association
TMF: transmitral flow
T2DM: type 2 diabetes mellitus
