# Supplementary material for: Effect of sitagliptin on the echocardiographic parameters of left ventricular diastolic function in patients with type 2 diabetes: a subgroup analysis of the PROLOGUE study
Source: Cardiovasc Diabetol. 2017 May 11;16:63. doi: 10.1186/s12933-017-0546-2 (PMC5426055; doi:10.1186/s12933-017-0546-2)
Supplement: Supplementary file 1 — Additional file 1. The PROLOGUE Study Investigators. [file 12933_2017_546_MOESM1_ESM.docx]

**Additional file 1. The PROLOGUE Study Investigators**

Masayoshi Ajioka (Department of Cardiovascular Internal Medicine, Tosei General Hospital); Toru Aoyama (Cardiology Center, Nagoya Kyoritsu Hospital); Tetsuya Babazono (Department of Medicine, Diabetes Center, Tokyo Women’s Medical University School of Medicine); Kazuoki Dai (Department of Cardiology, Hiroshima City Hospital); Jun Fukui (Division of Cardiology, Hokusho Central Hospital); Kumiko Hamano (Department of Diabetes and Endocrinology, Kanto Rosai Hospital); Shigemasa Hashimoto (Department of Cardiology, Karatsu Red Cross Hospital); Kazunori Hayashi (Department of Cardiology, Nakatsugawa Municipal Hospital); Yukihito Higashi (Department of Cardiovascular Regeneration and Medicine, Research Institute for Radiation Biology and Medicine, Hiroshima University); Tsutomu Hirano (Department of Diabetes, Metabolism, and Endocrinology, Showa University School of Medicine); Hideki Horibe (Department of Cardiovascular Medicine, Gifu Prefectural Tajimi Hospital); Kazuo Ibaraki (Department of Internal Medicine, Karatsu Red Cross Hospital); Masato Iida (Department of Cardiology, Mitsubishi Nagoya Hospital); Takako Iino (Department of Cardiovascular and Respiratory Medicine, Akita University Graduate School of Medicine); Kenji Iino (Department of Cardiovascular and Respiratory Medicine, Akita University Graduate School of Medicine); Teruo Inoue (Department of Cardiovascular Medicine, Dokkyo Medical University); Yutaka Ishibashi (Department of General Medicine, Shimane University Faculty of Medicine); Yuko S. Ishiguro (Department of Cardiology, Mitsubishi Nagoya Hospital); Masaharu Ishihara (Division of Cardiovascular Medicine and Coronary Heart Disease, Hyogo College of Medicine); Ryoji Ishiki (Division of Internal Medicine, Toyota Memorial Hospital); Tomoko Ishizu (Department of Clinical Laboratory Medicine, Faculty of Medicine, University of Tsukuba); Hiroshi Ito (Department of Cardiovascular and Respiratory Medicine, Akita University Graduate School of Medicine); Yoshito Iwama (Department of Cardiology, Meijo Hospital); Hideo Izawa (Department of Cardiology, Fujita Health University Banbuntane Hotokukai Hospital); Kohei Kaku (Department of Internal Medicine, Kawasaki Medical School); Kenshi Kan (Division of Diabetes, Metabolism and Endocrinology, Tokyo Medical University Hospital); Naoki Kashihara (Department of Nephrology and Hypertension, Kawasaki Medical School); Akira Kimura (Department of Cardiology, Meijo Hospital, Federation of National Public Service Personnel Mutual Aid Association); Ichiro Kishimoto (Department of Atherosclerosis and Diabetes, National Cerebral and Cardiovascular Center); Kazuo Kitagawa (Department of Neurology, Tokyo Women’s Medical University); Masafumi Kitakaze (Department of Clinical Medicine and Development, National Cerebral and Cardiovascular Center); Tomoki Kitano (Department of Cardiology, National Hospital Organization Nagoya Medical Center); Yoshihisa Kizaki (Department of Cardiology, Sasebo Chuo Hospital); Kenji Kohara (Division of Diabetes, Endocrinology and Metabolism, Kawasaki Medical School); Hiroshi Koiwaya (Department of Cardiology, Miyazaki Medical Association Hospital); Taizo Kondo (Department of Cardiology, Gifu Prefectural Tajimi Hospital); Toshimitsu Kosaka (Department of Cardiovascular and Respiratory Medicine, Akita University Graduate School of Medicine); Nehiro Kuriyama (Department of Cardiology, Miyazaki Medical Association Hospital); Shigetaka Kuroki (Eguchi Hospital); Koji Maemura (Department of Cardiovascular Medicine, Graduate School of Biomedical Sciences, Nagasaki University); Tatsuya Maruhashi (Department of Cardiovascular Medicine, Graduate School of Biomedical and Health Sciences, Hiroshima University); Hiroaki Masuzaki (Second Department of Medicine, Division of Endocrinology, Diabetes and Metabolism, Hematology, Rheumatology, Graduate School of Medicine, University of the Ryukyus); Kaori Miwa (Department of Neurology and Stroke Center, Osaka University Graduate School of Medicine); Takashi Miwa (Department of Diabetes, Endocrinology, Metabolism and Rheumatology, Tokyo Medical University); Tetsuro Miyazaki (Department of Cardiovascular Medicine, Juntendo University School of Medicine); Kazutaka Mori (Department of Cardiology, Nagoya Medical Center); Tomoatsu Mune (Division of Diabetes, Endocrinology and Metabolism, Kawasaki Medical School); Ikue Nakadaira (Diabetes and Endocrinology, Kanto Rosai Hospital); Mashio Nakamura (Department of Cardiology and Nephrology, Mie University Graduate School of Medicine); Yoshihito Nakashima (Department of Cardiovascular Disease, Tosei General Hospital); Masayuki Nakayama (JCHO Saga Central Hospital); Kosaku Nitta (Department of Medicine, Kidney Center, Tokyo Women’s Medical University); Yasunori Oguma (Department of Cardiovascular and Respiratory Medicine, Akita University Graduate School of Medicine); Hirotoshi Ohmura (Department of Cardiovascular Medicine, Juntendo University Graduate School of Medicine); Jun Ohno (Department of Cardiology, Tsushima Municipal Hospital); Shinji Okubo (Japan Labour Health and Welfare Organization, Kashima Hospital, Special Department and Cardiology, Tokyo Medical University); Jun-ichi Oyama (Department of Cardiovascular Medicine, Saga University); Sosho Ri (Division of Diabetes, Metabolism and Endocrinology, Internal Medicine Center, Showa University Koto Toyosu Hospital); Kenji Sadamatsu (Department of Cardiology, Saga Medical Center Koseikan); Makoto Saitoh (Department of Internal Medicine, Nishio Municipal Hospital); Masaki Sakakibara (Department of Cardiology, Handa City Hospital); Hiroaki Sano (Department of Cardiology, Nagoya Ekisaikai Hospital); Yasunori Sato (Department of Global Clinical Research, Graduate School of Medicine, Chiba University); Yoshisato Shibata (Department of Cardiology, Miyazaki Medical Association Hospital); Toshimasa Shigeta (Department of Cardiology, Gifu Prefectural Tajimi Hospital); Kenei Shimada (Department of Internal Medicine and Cardiology, Osaka City University Graduate School of Medicine); Fuji Somura (Department of Cardiology, Nagoya Central Hospital); Takashi Takei (Department of Medicine, Kidney Center, Tokyo Women’s Medical University); Toshiro Tanaka (Department of Internal Medicine, Nishio Municipal Hospital); Yoshito Tanioka (Division of Cardiology, Omura Municipal Hospital); Akihiro Terasawa (Department of Cardiology, Kasugai Municipal Hospital); Yasuo Terauchi (Department of Endocrinology and Metabolism, Yokohama City University); Hirofumi Tomiyama (Department of Cardiology, Tokyo Medical University); Masahiko Tsujii (Department of Gastroenterology, Osaka Rosai Hospital); Shuichi Tsuruoka (Department of Nephrology, Nippon Medical School); Shinichiro Ueda (Department of Clinical Pharmacology and Therapeutics, University of the Ryukyus); Hisashi Umeda (Division of Cardiology, Toyota Memorial Hospital); Mitsuyoshi Urashima (Division of Molecular Epidemiology, Jikei University School of Medicine); Hiroki Watanabe (Department of Internal Medicine, Nishio Municipal Hospital); Masato Watarai (Cardiovascular Center, Anjo Kosei Hospital); Takaaki Yamada (Department of Cardiology, Nagoya Medical Center); Hiroshi Yamamoto (Cardiovascular Surgery, Yamamoto Memorial Hospital); Akira Yamashina (Department of Cardiology, Tokyo Medical University); Kentaro Yamashita (Department of Cardiology, Nagoya University Graduate School of Medicine and National Hospital Organization Nagoya Medical Center); Takanori Yasu (Department of Cardiovascular Medicine, Dokkyo Medical University Nikko Medical Center); Chie Yasuoka (Department of Cardiovascular Medicine, Omura Municipal Hospital); Kiyoshi Yokoi (Department of Cardiovascular Medicine, Gifu Prefectural Tajimi Hospital).
